# Determination of a reliable assessment for occupational eye lens dose in nuclear medicine

**DOI:** 10.1002/acm2.13713

**Published:** 2022-07-01

**Authors:** Noriaki Miyaji, Kenta Miwa, Takashi Iimori, Kei Wagatsuma, Hiroyuki Tsushima, Noriyo Yokotsuka, Taisuke Murata, Tetsuharu Kasahara, Takashi Terauchi

**Affiliations:** ^1^ Department of Nuclear Medicine Cancer Institute Hospital of Japanese Foundation for Cancer Research Tokyo Japan; ^2^ Department of Radiological Sciences School of Health Sciences Fukushima Medical University Fukushima Japan; ^3^ Department of Radiology Chiba University Hospital Chiba Japan; ^4^ School of Allied Health Science Kitasato University Kanagawa Japan; ^5^ Department of Radiological Technology, Faculty of Health Sciences Kobe Tokiwa University Hyogo Japan; ^6^ Department of Radiological Technology, Faculty of Medical Technology Teikyo University Tokyo Japan

**Keywords:** dose equivalent, dosimeter location, eye lens, occupational exposure, radionuclide

## Abstract

The most recent statement published by the International Commission on Radiological Protection describes a reduction in the maximum allowable occupational eye lens dose from 150 to 20 mSv/year (averaged over 5‐year periods). Exposing the eye lens to radiation is a concern for nuclear medicine staff who handle radionuclide tracers with various levels of photon energy. This study aimed to define the optimal dosimeter and means of measuring the amount of exposure to which the eye lens is exposed during a routine nuclear medicine practice. A RANDO human phantom attached to Glass Badge and Luminess Badge for body or neck, DOSIRIS and VISION for eyes, and nanoDot for body, neck, and eyes was exposed to ^99m^Tc, ^123^I, and ^18^F radionuclides. Sealed syringe sources of each radionuclide were positioned 30 cm from the abdomen of the phantom. Estimated exposure based on measurement conditions (i.e., air kerma rate constants, conversion coefficient, distance, activity, and exposure time) was compared measured dose equivalent of each dosimeter. Differences in body, neck, and eye lens dosimeters were statistically analyzed. The 10‐mm dose equivalent significantly differed between the Glass Badge and Luminess Badge for the neck, but these were almost equivalent at the body. The 0.07‐mm dose equivalent for the nanoDot dosimeters was greatly overestimated compared to the estimated exposure of ^99m^Tc and ^123^I radionuclides. Measured dose equivalents of exposure significantly differed between the body and eye lens dosimeters with respect to ^18^F. Although accurately measuring radiation exposure to the eye lenses of nuclear medicine staff is conventionally monitored using dosimeters worn on the chest or abdomen, eye lens dosimeters that provide a 3‐mm dose equivalent near the eye would be a more reliable means of assessing radiation doses in the mixed radiation environment of nuclear medicine.

## 1 INTRODUCTION

The optimization and reduction of occupational exposure to radiation is an absolute principle in terms of radiation protection. Exposing the eye lens to radiation is a major concern for medical staff because it can cause lens opacity or cataracts.^1^ The International Commission on Radiological Protection (ICRP) publishes recommendations for radiation protection from expert perspectives. In addition, this organization has determined effective and equivalent doses as an alternative to dose equivalents to monitor occupational exposure based on accumulated epidemiological evidence. The ICRP recommends in ICRP 118 to reduce the annual occupational limit of equivalent radiation doses to the eye lens from 150 to 20 mSv (averaged over 5‐year periods) with no single year exceeding 50 mSv.^2^ This dose limit for the eye lens was incorporated into Japanese medical laws and actions associated with preventing radiation hazards in April 2021.

Nuclear medicine staff cannot avoid exposure to radiopharmaceutical agents that are prepared and administered to patients compared with staff who conduct general radiological examinations. The maximum annual eye dose exceeds 5 mSv in 20% of nuclear medicine staff in European hospitals,^3^ and exposure is in the 10–50‐mSv range in 20% of nuclear medicine departments.^4^ An American survey of occupational exposure found a significantly increased risk of cataracts among radiological technologists who had conducted at least one nuclear medicine procedure.^5^ Therefore, the International Atomic Energy Agency, which seeks to promote the safe, secure, and peaceful use of nuclear technologies, has described in their Tec Doc‐1731 that the new occupational dose limits for the eye lenses of nuclear medicine staff are likely to be exceeded.^6^

Current Japanese regulations define occupational exposure to nuclear medicine workers in terms of the whole body rather than in individual locations of the body. Passive dosimeters generally apply 10‐ and 0.07‐mm dose equivalents, namely, *H*_p_(10) and *H*_p_(0.07), respectively, at the closest attachment position to determine eye lens exposure.^7^ However, males and females wear dosimeters around the chest and abdomen, respectively. The estimated operational quantities of personal and ambient doses to the eye lens recommended by the International Commission on Radiation Units and Measurements (ICRU) is 3‐mm dose equivalents *H*_p_(3).^8^, ^9^ The *H*_p_(3) can be conservatively evaluated by measuring whichever is greater between *H*_p_(10) or *H*_p_(0.07). If a monitored radiation field is established (i.e., uniform), the *H*_p_(3) can also be estimated from passive dosimeter measurements based on empirical data from *H*_p_(3)/*H*_p_(10).^10^, ^11^, ^12^ However, the nuclear medicine environment comprises mixed radiation fields derived from multiple sources and various amounts of energy emitted due to administering patients with radionuclide tracers.^13^ The dose equivalent according to depth *H*_p_(*d*) is energy dependent. For instance, exposure to low‐energy photons is lower at *H*_p_(10) than that at *H*_p_(0.07) because the maximum peak point is shallow. Although the appropriate dose equivalent should be selected for the energy used, the measurement means of eye lens doses are not standardized. Eye lens doses estimated by a dosimeter attached to body and neck can include many potential factors of bias and variance such as dose equivalent, dosimeter location, energy and angle dependence, and radiation field geometry. Thus, radiation doses to the eye lenses of staff in nuclear medicine areas should be assessed using a specialized dosimeter located near the eye. In previous reports, the *H*_p_(3)/*H*_p_(10) using eye lens and body dosimeter has almost underestimated below 1.0 except for nuclear medicine staff of short attachment days.^14^, ^15^

Monitoring eye lens exposure in nuclear medicine has suggested assessment at *H*_p_(3).^13^ The Optimization of Radiation Protection of Medical Staff (ORAMED) project in Europe, which perform the international response to the revised ICRP recommendations, led to the development and validation of wearable dosimeters for the eye lens to appropriately measure values close to the eyes at *H*_p_(3).^16^ In 17 of 20 dosimeters, 90% of responses complied with the International Organization for Standardization (ISO), 14146 standard requirements.^17^ The performances of two types of Japanese dosimeters for eye lenses have been evaluated in the diagnostic X‐ray energy domain (interventional radiology, cardiology) and in staff at Fukushima Daiichi Nuclear Power Plant in Japan.^18^, ^19^, ^20^ However, the utility of eye lens dosimeters for nuclear medicine staff who handle radionuclide tracers with various levels of photon energy has remained unclear. An attenuation caused by personal protective equipment, dosimeters, and body type can significantly differ among combinations of high‐ and low‐energy photons; thus, dose variations might be large.

Phantoms are useful and essential to define the characteristics of these dosimeters that cannot be compared in clinical studies because they can be constructed with known materials and desirable radionuclides. We compared five types of Japanese passive dosimeters using a phantom to determine a reliable assessment for occupational eye lens dose.

## 2 MATERIALS AND METHODS

### 2.1 Dosimeters

**FIGURE 1:**
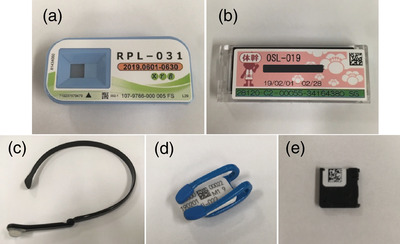
Manufactured dosimeters: (a) Glass Badge, (b) Luminess Badge, (c) DOSIRIS, (d) VISION, and (e) nanoDot

We selected five dosimeters that use different technologies and principles (Figure 1). Table 1 lists the main technical features of each dosimeter. Glass Badge (Chiyoda Technol Co. Ltd., Tokyo, Japan) and Luminess Badge (Nagase‐Landauer, Ltd., Tsukuba, Japan) dosimeters (neck or body dosimeter) with various filters are prevalent among medical staff in Japan. Both dosimeters can be worn at the neck, chest, and abdomen and estimate the *H*_p_(0.07) or *H*_p_(10) from an amount of luminescence of dosimeter element. In contrast, the VISION (Nagase‐Landauer, Ltd.) and DOSIRIS (Chiyoda Technol Co. Ltd., Tokyo, Japan) dosimeters for eye lenses (eye lens dosimeter) are placed on a narrow headband adjacent to or near the eyes or on personal protective eyewear.^17^, ^21^ This allows measurement at only *H*_p_(3), which is the appropriate depth with which to estimate eye lens doses. A small optically stimulated luminescence (OSL) dosimeter, namely, “nanoDot” (Nagase‐Landauer, Ltd.), can obtain *H*_p_(0.07). Although this is generally used to confirm skin dose measurements in patients, it can also monitor eye lens exposure because *H*_p_(3) can be determined using a conversion factor.^22^ Dosimetry services in the USA and Thailand proposed the nanoDot dosimeter to measure eye lens exposure among clinical staff.^12^ We measured and analyzed the results of nanoDot placed at the eye, neck, chest, and abdomen.

**TABLE 1:** Main technical features of dosimeters

	Dosimeter				
Type	Body, neck		Eye lens		Skin
Feature	Glass Badge	Luminess Badge	DOSIRIS	VISION	nanoDot
Element	RPL	OSL	TLD	TLD	OSL
Filter	Plastic, Al, Cu, Sn	Plastic, Al, Ti, Sn	None	None	None
Range of photon energy (MeV)	0.01–10	0.005–20	0.024–1.25	0.016–1.25	0.005–20
Attachment position	Neck, chest, abdomen		Eye		All
Calculated dose equivalent	*H*_p_(10), *H*_p_(0.07)		*H*_p_(3)		*H*_p_(0.07)
Size (mm)	60 × 28.3 × 7.6	72 × 44 × 10	59 × 16 × 4	23 × 13 × 8	10 × 10 × 2

Abbreviations: Al, aluminum; Cu, copper; OSL, optically stimulated luminescence; RPL, radio photoluminescence; Sn, tin; Ti, titanium; TLD, thermo‐luminescence dosimeter.

### 2.2 Reproducibility of dosimeters in air

We evaluated the quantitative reproducibility of 25 dosimeters (*n* = 25 each) in air without scatter objects. All samples except the nanoDot were analyzed using readers specific to each manufacturer. The Glass Badge and Luminess Badge dosimeters were applied *H*_p_(10) dose equivalent. As the detection limit of the dosimeter is around <0.1 mSv, the duration of irradiation was two‐ to threefold above the detection limit to reduce measurement uncertainty. All dosimeters were positioned at 30 cm from a ^99m^Tc source to which the phantom was continuously exposed for 24 h. All dosimeters were calibrated using a traceable ^137^Cs (662 keV) source, which was close to the energy range used by a nuclear medicine, based on the Japanese Standards Association (JIS) Z 4345‐3, including an uncertainty measurement. The JIS, which has harmonized with ISO, is established as a national standard according to the Japanese Standardization Act. All dosimeters were tested and irradiated according to ISO 4037‐3.^23^, ^24^ All nanoDots, dosimeters including a control, were consecutively assessed *H*_p_(0.07) three times using a microStar reader (Nagase‐Landauer, Ltd.). Three reads from control nanoDot dosimeters that were not exposed to radionuclides were averaged and subtracted from the exposed nanoDot dosimeters to obtain a single data point. The coefficients of variance (CV) of the measured doses of the exposed dosimeters were calculated as

$$\%\mathrm{CV}\;=\left(\mathrm{SD}/D_{\mathrm{ave}}\right)\;\times 100,$$

where SD is the standard deviation of the measured dose, and *D*_ave_ is the average exposure measured by each dosimeter.

### 2.3 Phantom study with dosimeter

**FIGURE 2:**
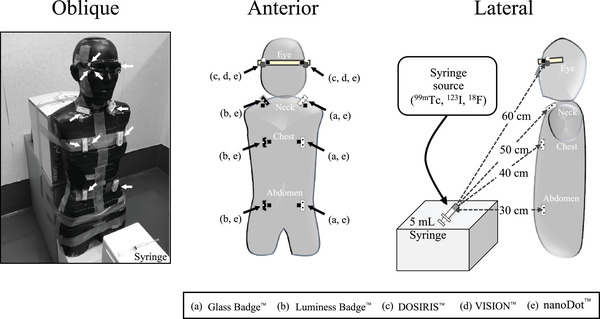
Locations of five types of dosimeters in a phantom exposed to ^99m^Tc. Oblique, anterior, and lateral views of phantom with all dosimeters attached. Eye (c, d, e): DOSIRIS, VISION, and nanoDot, respectively. Neck, chest, and abdomen (a, b, e): Glass Badge, Luminess Badge, and nanoDot, respectively

Each dosimeter was attached to the adult male human tissue‐equivalent RANDO phantom (The Phantom Laboratory, New York, NY, USA) to simulate clinical eye, neck, chest, and abdominal locations. We consecutively exposed the phantom to ^99m^Tc, ^123^I, and ^18^F radionuclides diluted in the 3‐ml water in 5‐ml syringes for 24, 51, and 3 h, respectively. The amount of exposure to each radionuclide was determined in triplicate. The source shape does not affect the equivalent dose of the eye lens if the distance is >30 cm.^25^ Nuclear medicine procedures have a high risk of occupational abdominal exposure while dispensing and administering radionuclides to patients.^26^ Thus, the syringe source was positioned at 30 cm from the abdomen of the phantom. Each type of dosimeter was attached to the chest, neck, and eye at 40, 50, and 60 cm, respectively, from each source (Figure 2).

**FIGURE 3:**
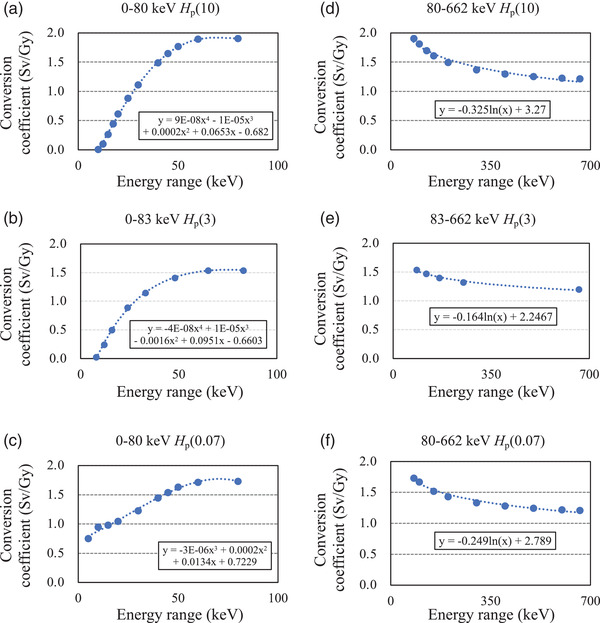
Conversion coefficients for X and γ rays. Blue point: conversion coefficient for X and γ rays energy to *H*_p_(10), *H*_p_(3), *H*_p_(0.07) for a slab and pool phantom based on Japanese Standards Association (JIS) Z 4345‐3. Parts (a–c) and (d–f), respectively, indicate low‐ and high‐energy photon in each dose equivalent. Blue dotted line: polynomial interpolation or logarithmic approximation based on conversion coefficient

These results were normalized to 30 cm from the source to clarify differences among the dosimeters according to the square of the distance from it. If the distance exceeded >30 cm, irradiation doses are uniform independently of the source shape.^25^ The dose equivalent of Glass Badge and Luminess Badge dosimeters was used at *H*_p_(10) because the energy band for nuclear medicine is adopted *H*_p_(10) rather than *H*_p_(0.07) in principle. The exposure was analyzed in terms of the position, type of radionuclide, and type of dosimeter. We calculated a measurement error between the measured and the estimated dose equivalents as

$$\mathrm{Measurement}\;\mathrm{error}\left(\%\right)=\left(1-\left(D_{m}-D_{e}\right)\right)/D_{e}\times 100,$$

where *D*_m_ is the mean dose equivalent of three measurements per dosimeter, and *D*_e_ is the estimated dose equivalent calculated using an air kerma rate constant and the measurement conditions in Table 2. Figure 3 shows the relevant assumptions of the conversion coefficient. The conversion coefficient per photon energy of the dose equivalent for each dosimeter was calculated, and each main energy was applied by polynomial interpolation based on the Behrens and JIS reports.^24^, ^27^ The exposure rate constant is defined as the ratio of the product of the exposure rate and the square of the distance from the source to a dosimeter.

**TABLE 2:** Conditions under which doses of the three radionuclides were estimated

	Main photon energy (keV)	Distance (m)	Dose equivalent (Sv)	Air kerma rate constant (μSv m^2^/MBq h)	Conversion coefficient (Sv/Gy)	Activity (MBq)	Reduction factor	Exposure time (h)	Half time (h)	Estimated dose (μSv)
^99m^Tc	141, 18.3, 20.6	0.3	*H*_p_(10)	0.0141	1.58	982	0.336	24	6.01	1956.0
			*H*_p_(3)		1.37					1702.6
			*H*_p_(0.07)		1.52					1880.6
^123^I	529, 159, 31.7, 31.0, 27.5, 27.2, 3.8	0.3	*H*_p_(10)	0.0361	1.38	271	0.347	51	13.27	2658.2
			*H*_p_(3)		1.18					2264.7
			*H*_p_(0.07)		1.35					2600.7
^18^F	511	0.3	*H*_p_(10)	0.1351	1.24	780	0.593	3	1.83	2589.5
			*H*_p_(3)		1.22					2549.4
			*H*_p_(0.07)		1.24					2574.9

### 2.4 Statistical analysis

All data were statistically analyzed using Prism 6 v. 6.0 (GraphPad Software Inc., San Diego, CA, USA). Differences in mean dose equivalents between eye lens and body dosimeters were compared using Mann–Whitney *U* tests. To simulate Japanese eye dose monitoring, we combined the chest (male) and abdomen (female) measurements into a single data point. Although the effects of each location of nanoDot were evaluated, we excluded neck data from dosimeter comparisons. Measured dose equivalents with *p* < 0.05 were considered significant.

## 3 RESULTS

Table 3 shows the %CV of each dosimeter in air. The %CV of <5.0%, derived from the eye lens dosimeters DOSIRIS and VISION indicated good reproducibility. The CV was <8.0% for all dosimeters. Among the dosimeters, the mean ± SD of the Luminess Badge was 1.21 ± 0.09 μSv and the CV was appreciably higher than that of the other dosimeters. In contrast, the mean ± SD of the nanoDot was 1.80 ± 0.03, with the most stable CV of 1.52%.

**TABLE 3:** The reproducibility of the amount of measured dose equivalent (mSv) by each dosimeter in air

	Dosimeter				
	Glass Badge	Luminess Badge	DOSIRIS	VISION	nanoDot
1	1.44	1.07	1.85	1.32	1.77
2	1.48	1.30	1.86	1.29	1.79
3	1.44	1.19	1.96	1.29	1.82
4	1.39	1.23	1.86	1.21	1.83
5	1.29	1.24	1.92	1.19	1.77
Average	1.41	1.21	1.89	1.26	1.80
SD	0.07	0.09	0.05	0.06	0.03
%CV	5.20	7.10	2.54	4.49	1.52

Abbreviations: %CV, percentage coefficient of variation; SD, standard deviation.

**FIGURE 4:**
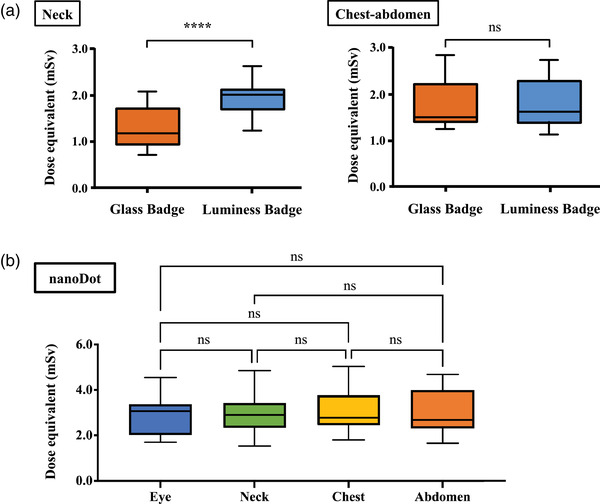
Comparison of dose equivalents (mSv) measured using Glass Badge and Luminess Badge. Glass Badge and Luminess Badge were placed on neck and chest–abdomen (a), and nanoDot (b) was placed at all locations. Asterisks indicate significant differences (^****^ *p* < 0.001)

Figure 4 shows box‐and‐whisker plots of locations of Glass Badge, Luminess Badge (Figure 4a), and nanoDot (Figure 4b) dosimeters. The dose equivalent *H*_p_(10) at the neck was significantly lower for the Glass Badge than for the Luminess Badge (*p* = 0.0009), whereas there was almost identical between them at the chest–abdomen location (Figure 4a). In contrast, the dose equivalent *H*_p_(0.07) at any tested location did not significantly differ for the nanoDot (Figure 4b).

**FIGURE 5:**
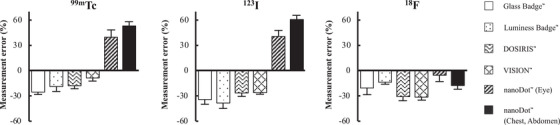
A measurement error (%) compared with dose equivalents estimated using five types of dosimeters. A measurement error is based on mean dose equivalents for ^99m^Tc, ^123^I, and ^18^F

Figure 5 shows the measurement errors of the dosimeters for the three radionuclides. The measurement error of the nanoDot for ^99m^Tc and ^123^I tended to be overestimated, whereas those of all dosimeters for ^18^F were underestimated compared with the estimated dose.

**FIGURE 6:**
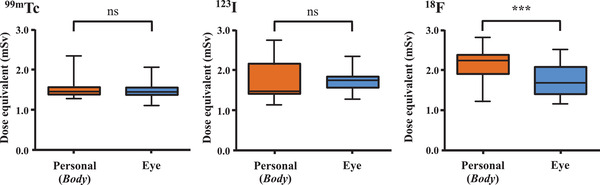
Comparison of dose equivalents (mSv) of three radionuclides between body and eye lens (*Eye*) dosimeters. The dosimeter attached to the body and eye lens dosimeter, respectively, showed a combined data point of Glass Badge and Luminess Badge, DOSIRIS, and VISION. Asterisks indicate significant differences (^***^ *p* < 0.005)

Figure 6 shows box‐and‐whisker plots of differences in radiation exposure determined by each dosimeter attached to body and eyes according to the three radionuclides. These results of attached dosimeter to body and eyes dosimeter were combined data point of Glass Badge and Luminess Badge, DOSIRIS, and VISION, respectively. The dose equivalents determined by the Glass Badge and Luminess Badge, DOSIRIS, and VISION for ^99m^Tc and ^123^I were similar and did not significantly differ (*p* = 0.0049). In contrast, exposure to ^18^F was significantly lower for the eye lens dosimeter than body dosimeter.

## 4 DISCUSSION

The occupational exposure of nuclear medicine staff to radiation should be adequately and reasonably managed in accordance with the safety principle of, “as low as reasonably achievable.” We determined a reliable method of assessing occupational eye lens doses by comparing the DOSIRIS and VISION for eye lens, the Glass Badge and Luminess Badge positioned at the neck and body, and the nanoDot dosimeter by ^99m^Tc, ^123^I, and ^18^F radionuclides. We found that the dosimeter attached to neck decreased reproducibility. The amount of exposure to ^18^F significantly differed between body dosimeter *H*_p_(10) and eye lens dosimeter *H*_p_(3). These dosimeters attached to body or neck are affected by the locations as this influences incidence angles and irradiation distance because these differ the design and technology. Although the nanoDot overestimated exposure of the eye lens compared with others for ^99m^Tc and ^123^I, these dosimeters with protective glasses might be more accurate than body dosimeters. The DOSIRIS and VISION that can evaluate *H*_p_(3) near the eye would deliver more reliable results than others because the conversion from *H*_p_(0.07) or *H*_p_(10) to *H*_p_(3) for Glass Badge, Luminess Badge, and nanoDot dosimeter can be varied according to the type of radiation. How to wear such dosimeters is described in the new Japanese guidelines for monitoring eye lens exposure.^28^ Although the *H*_p_(3) in air measured by ^99m^Tc varied within ±5.0% between these dosimeters, *H*_p_(3) values measured using the phantom were almost identical. The conversion coefficient for air kerma into personal dose equivalents is generally determined using passive dosimeters and a slab phantom (dimensions: 30 × 30 × 15 cm^3^).^29^ Only DOSIRIS and VISION dosimeters have been assessed using a cylinder phantom simulating the head. The ORAMED project found that different amounts of scatter between the slab and cylinder phantoms significantly impacted the conversion coefficient in the low‐energy range.^30^ Our findings indicated that the dose equivalents estimated by dosimeters considerably varied not because of scatter, but rather because of differences in a phantom calibration.

One of the major concerns of dosimeters attached to body and neck is their potential angular dependence.^31^, ^32^ The most popular dosimeter standard in Japan is JIS Z 4345 (2017) based on ISO 4037‐3, which allows variations of 0.71–1.67 in angles ranging from 0° to 60°.^24^ Da Silva et al. also indicated that an angular variation of ±35% for incidence angles up to 60° complies with the ISO 4037 criteria.^33^ The *H*_p_(10) was significantly lower when measured by the Glass Badge than the Luminess Badge at the neck, whereas those located at the body were almost equivalent (Figure 4a). In contrast, data measured by a small nanoDot without a filter did not significantly differ among locations (Figure 4b). These dosimeters wearing along the clavicle have poor reproducibility caused by differences in three‐dimensional orientation such as the oblique attachment orientation and the angle of incident γ rays. We concluded that the symmetrical neck attachment of the Glass Badge and Luminess Badge could not be reproduced due to measurement bias inherent in their design and principles. The difference between neck and body attachment significantly affects the *H*_p_(10) of these dosimeters because of exposure to detector elements without passing through an appropriate filter. Fujibuchi et al. showed that dosimeters attached to the neck, chest, and abdomen result in nonuniform organ‐absorbed doses.^34^ We considered this sort of bias occurred among the dosimeters in the present study because we assessed the effects of placing them to simulate typical monitoring in Japan. Thus, estimating exposure to eye lenses with such dosimeters worn around the neck can be unreliable for Japanese nuclear medicine staff, who mainly handle radioactivity sources near the abdomen.

Nuclear medicine staff are exposed to various fields of photons, electrons, and positrons over a broad energy range. Thermoluminescent dosimeters (TLD) to monitor the eye lens can reduce energy fluctuations better than the OSL element because of the low intrinsic energy dependence of the TLD element.^35^ VISION, DOSIRIS with TLD, and Glass Badge, Luminess Badge with the function of energy discrimination can provide stable responses from low to high energy.^36^ In contrast, the nanoDot, which has an Al_2_O_3_‐based OSL element without a filter and is calibrated by ^137^Cs, overestimates *H*_p_(0.07) with respect to water or tissue by a factor of ∼4.0 in the diagnostic X‐ray energy domain of 50–110 keV.^37^ Although ^18^F emits only 511‐keV annihilation photons, ^99m^Tc emits characteristic X‐rays (emission probabilities) of 18.3 (6.2%) and 20.6 (1.1%) keV, and ^123^I emits characteristic X‐rays of 27.5 (45.6%), 27.2 (24.7%), 3.8 (9%), 31.0 (8.1%), 30.9 (4.2%), and 31.7 (2.3%) keV, respectively.^38^ The nanoDot, a single element of *H*_p_(0.07) assessment, influences measured values of characteristic X‐rays such as ^99m^Tc and ^123^I even at lower emission probabilities, which is clear in Figure 5. The nanoDot with the energy dependence of OSLDs and a dose response was increasingly prominent with decreasing energy where the highest divergence factor was 3–4. This aspect should be considered being specific to not only unique to nuclear medicine but also to other imaging modalities, such as dual energy CT and scattered X‐ray radiation in IR/F. As a technical method of solving the problem, the use of radiation protection products that can eliminate low energy components might be the most reproducible of the nanoDot. Although protective glasses cannot shield against ^18^F annihilation photons,^39^ wearing protective glasses in nuclear medicine can reduce external exposure to ^99m^Tc and ^123^I.^40^ The lead glasses should be worn when monitoring exposure using the nanoDot can be exceeding the maximum eye dose. Exposure to ^18^F was significantly higher to a body dosimeter than to eye lens dosimeters (Figure 6). Kopec et al. also indicated the *H*_p_(3) is significantly lower when measured using around eyes attachment, compared with the *H*_p_(10) of a body attachment in clinical PET facilities.^14^ A new method that estimated *H*_p_(3) with measured values of *H*_p_(10) and *H*_p_(0.07) on body has been suggested, but differential transmissions of γ, α, and β emitters influence each dose equivalent. Although the nanoDot measured dose equivalent *H*_p_(0.07) can be also the accurate assessment, eye lens monitoring using conversion factors to estimate *H*_p_(3) is poor reliability under the environment of different radiation types. Consequently, *H*_p_(3) recommend to monitor by DOSIRIS and VISION attached near the eye.^41^ The direct monitoring at a 3‐mm depth will become increasingly important for nuclear medicine staff who handle radionuclide tracers with variable photon energy emission.

This study has some limitations. The baseline for comparable tests is typically assumed as “ground truth,” such as ion chamber dose measurements, rigorous dose calculations, or Monte Carlo simulations, for relative comparisons (overestimated, underestimated) to be meaningful. Our estimated dose “*D*_e_” might have uncertainty in terms of the true dose due to being calculated based on the main photon emission of the cited reference. We analyzed data generated using a phantom that simulated a human body wearing dosimeters. Further clinical validations of cyclotron operators, technologists, nurses, and doctors are required to achieve reliable measurements, exposure, and operational aspects for eye lens monitoring. In addition, the impact of radiation exposure on therapeutic radionuclides emitting α, β, and γ rays caused by decay remains unclear because dosimeters have been validated based on only three diagnostic radionuclides applied in nuclear medicine.^42^

## 5 CONCLUSIONS

We evaluated eye lens exposure using a phantom simulating nuclear medicine staff wearing various dosimeters. The measurement accuracy of occupational exposure for eye lenses using dosimeters was affected by various factors such as the type and design of dosimeters, where they are worn, and radionuclides. Although radiation exposure to the eye lens of nuclear medicine staff is conventionally monitored by dosimeters worn around the chest or abdomen, we recommend the eye lens dosimeter to reduce the uncertainty of measurements in terms of reproducibility, angular dependence, dose equivalent, and energy. We suggest that staff wear a nanoDot dosimeter inside a radiation protection product to eliminate the low energy domain. Our findings provide useful information regarding the reliable assessment of radionuclide doses to the eye lens.

## CONFLICT OF INTEREST

The authors declare that there is no conflict of interest that could be perceived as prejudicing the impartiality of the research reported.

## AUTHOR CONTRIBUTIONS

Kenta Miwa, Noriaki Miyaji, and Takashi Terauchi designed the study. Noriaki Miyaji, Takashi Iimori, Kei Wagatsuma, Hiroyuki Tsushima, Noriyo Yokotsuka, Taisuke Murata, and Tetsuharu Kasahara collected the data. Kenta Miwa and Noriaki Miyaji interpreted the data. Kenta Miwa and Noriaki Miyaji drafted and revised the manuscript. All authors read and approved the final version of the manuscript.
